# A new performance analysis method for rolling bearing based on the evidential reasoning rule considering perturbation

**DOI:** 10.1038/s41598-022-21885-y

**Published:** 2022-10-25

**Authors:** Yunyi Zhang, Guohui Zhou, Wei Zhang, Wei He, Yuhe Wang, Yizhe Zhang, Peng Han

**Affiliations:** 1 Harbin Normal University, Harbin, 150025 China; 2 Rocket Force University of Engineering, Xi’an, 710025 China

**Keywords:** Computational science, Computer science

## Abstract

Rolling Bearing is a key component of the transmission of rotating machinery, and it is widely used in industrial fields. Therefore, it is of vital importance to evaluate the performance and reliability of rolling bearing. Aiming at the interference problems faced by rolling bearings during operation, a performance evaluation model based on the evidential reasoning (ER) rule is proposed in this article. Firstly, the time domain and frequency domain characteristic indicators of bearing vibration signals are taken as evaluation indicators, and the evaluation system is constructed. Secondly, various indicator information is unified into a belief structure, and the reliability and the weight of the indicators are fully considered in the ER rule. Thirdly, to simulate the complex working environment of rolling bearings, the perturbation analysis method is adopted. After determining the maximum perturbation error and perturbation coefficient, the performance reliability of the rolling bearing is analysed, and a performance reliability evaluation model considering perturbation is proposed. Finally, based on the whole-life open data set of rolling bearing from the University of Cincinnati, the validity and reliability of the proposed model are verified in performance analysis.

## Introduction

Rolling bearing is a precise mechanical element that converts the sliding friction between the running shaft and the shaft seat into rolling friction, thereby reducing friction. It is commonly used as a rotating part and the main supporting part in the rotating device. It is widely used in machinery^1^, aviation^2^, transportation^3^ and other fields. Rolling bearing have the characteristics of high running accuracy, low power loss, and limited adaptability to working conditions. Based on the above characteristics, environmental interference, load intensity, and self-wear can easily disturb the bearing performance, so it is essential to study the bearing performance state and performance reliability under perturbation conditions.

For the performance evaluation of rolling bearing, many researches have been given by scholars at home and abroad. At present, the commonly used methods of performance evaluation and reliability analysis include model-driven approaches, data-driven approaches and deep learning approaches. For model-driven approaches, Tran et al. studied mechanical performance degradation assessment based on equipment proportional failure models and support vector machines^4^. Cubillo et al. proposed a combination of data-driven technology and physics-based technology to establish a rolling bearing’s performance degradation model, evaluate the state of hydrodynamic bearing and achieve good results^5^. Ding et al. used the proportional failure rate model as reliability evaluation model, and the effective value and kurtosis were used as the model covariates to evaluate the reliability of railway locomotive bearing in real time^6^. Gao et al. proposed a prediction method of rolling bearing operational reliability based on isometric mapping and a nonhomogeneous cuckoo search-least squares support vector machine (NoCuSa-LSSVM). To reduce the dimension of high-dimensional acquisition, which is composed of time domain, frequency domain and time–frequency domain features of vibration signals, the isometric mapping algorithm is adopted. The NOCUSA-LSVM model is used to predict the state characteristics of bearing performance degradation, which can obtain the prediction results of bearing operating reliability^7^. Yang et al. established a novel RUL prediction model based on degradation indicators. The vibration signal of the rolling bearing is decomposed into some intrinsic scale components, and the time break point is obtained by signal reconstruction. The generalized regression neural network model based on HI is constructed to predict the bearing’s RUL^8^.

From the perspective of data-driven approaches, Qin et al. proposed a digital twin model of a rolling bearing driven by a data-model combination, and a life-cycle bearing dynamic model in virtual space was formed. By comparing the obtained digital twin result with the measured signal in the time domain and frequency domain, the effectiveness of the model was verified^9^. Wu et al. proposed a degradation condition monitoring method based on adaptive noise and fully integrated empirical mode decomposition and principal component analysis for rolling bearing. After analyzing the robustness and attenuation of health indicators, it can identify the running state of rolling bearings^10^. Wang et al. constructed a data-driven rolling bearing fault detection model based on vibration signal analysis, realizing fault detection and early warning^11^. Wu et al. proposed an evaluation method of bearing performance degradation based on fuzzy C-means clustering, by studying the process of data-driven performance degradation to evaluate the rolling bearing’s degradation state^12^.

From the perspective of deep learning approaches, Jaouher et al. used a data-driven method to study bearing life prediction based on Weibull and artificial neural network^13^. Jin et al. proposed an intelligent method to achieve the contact fatigue reliability analysis of aviation bearing and applied an artificial neural network to achieve fatigue reliability analysis^14^. To reflect the health state of rolling bearings, Wang et al. proposed a performance degradation evaluation architecture, which is based on the Internet and deep long short-term memory^15^. Based on the convolutional neural network and bidirectional long short-term memory network model, Cheng et al. proposed a new rolling bearing health prediction method, which can predict the remaining life of rolling bearing by predicting nonlinear degradation indicators^16^.

The above performance analysis of rolling bearing focuses on model-driven, data-driven and deep learning. The model-driven performance evaluation method needs to establish a bearing performance evaluation model accurately. However, the mechanism of performance degradation process is complex in engineering, so it is difficult to establish a universal evaluation model of all kinds of rolling bearings. The data-driven performance evaluation approach uses algorithms to mine performance degradation information from historical monitoring data^17,18^. However, the objectivity of data-driven approaches is strong and the subjective control of expert knowledge is ignored. Deep learning approaches often use neural networks to evaluate the performance, but the evaluation process is in a black box, which is not interpretable. Moreover, the real-time rolling bearing data are unsupervised. General unsupervised learning methods, such as knowledge-based analytic hierarchy processes and data-based clustering methods, are prone to interference from extreme data and cannot effectively consider perturbations. To evaluate the performance of rolling bearing, multiple performance characteristics and perturbations should be comprehensively considered. The above methods have no good effect on evaluating the performance of rolling bearing under perturbations. The ER rule has a good processing effect in uncertainty and multi-index joint reasoning. In addition, the ER rule and perturbation analysis can be combined^27^, so we used the ER rule to evaluate the performance of rolling bearing in this article.

In 2013, Yang et al. established the ER rule considering evidence weight and evidence reliability. The ER rule is a further development and extension of Dempster–Shafer (D–S) theory^19^ and the ER algorithm^20^. It clearly distinguished the importance and reliability of evidence, and forms a general joint probabilistic reasoning process. It can effectively solve problems such as evidence conflict and index explosion in D–S evidential reasoning^21^. In the ER rule, weight and reliability are fully considered, and experts’ subjective experience and objective data are combined to describe the data, which has great advantages in dealing with information uncertainty^22,23^. The ER rule shows good effect on uncertainty and considers the evidence weight and the evidence reliability. Therefore, it has been widely used in many fields such as evaluation and decision making. The ER rule is a semi-quantitative evaluation method, semi-quantitative evaluation method can evaluate the objective from qualitative and quantitative aspects^24,25^, considering subjectivity and objectivity, and making the evaluation result reasonable. For example, Zhao et al. proposed an online security evaluation method based on evidential reasoning, which integrates the state of the system at “history”, “current” and “future” moments to evaluate the comprehensive security level of the system^26^. Zhou et al. extended the ER rule to the MADM problem in a group decision-making environment. In the process of evaluating the service life of electric vehicles, interval weights, reliability of experts and evidence are fully utilized^27^. Xu et al. applied the ER rule to the fusion decision problem of uncertain fault feature information and achieved good results in fault diagnosis of motor rotors^28^. The above cases demonstrate the effectiveness of the ER rule in the evaluation and decision-making fields. Based on the advantages of the ER rule in dealing with uncertainty and fuzzy information, it is used to evaluate the performance state of the rolling bearing in this article.

Due to rolling bearings are easily affected by their own and external disturbances in operation, there are no specific methods to evaluate its performance under perturbation conditions. Therefore, to analyse the adaptability of bearing performance to different disturbances, the perturbation analysis method is used to simulate internal and external disturbances. Perturbation analysis was proposed by Ho. It was initially applied to a discrete event dynamic system by adding a disturbed sample track on the basis of original data, and it was used to analyse the sensitivity of the system performance index to a key parameter^29^. Tang et al. proposed the ER rule considering perturbation and verified the practicability of this method^30,31^. The above literature provides a theoretical basis for the research and application of the ER rule and perturbation analysis, which can be applied to the reliability analysis of rolling bearing performance.

In summary, the bearing performance evaluation model based on the ER rule and performance reliability analysis model considering perturbation are constructed in this article. The contributions of this article are described as follows:

1. The time domain and frequency domain characteristics of rolling bearing vibration signals are used as evaluation indicators to construct an evaluation factor system.
2. The ER rule can effectively fuse multiple pieces of information, which is suitable for fusing multiple characteristic indicators of bearing vibration signals to obtain reasonable performance evaluation results. Therefore, it is used to evaluate the performance state of rolling bearing, which can clearly show the performance level of rolling bearing at different times.
3. The perturbation analysis method is used to simulate the internal and external perturbations during the operation of rolling bearing. The adaptability of bearing to perturbations is quantified.

The rest of this article is organized as follows: the performance and performance reliability of rolling bearing are described in “Problem definition”, and all the steps of the evaluation process are determined. The evaluation indicators, evaluation model of the ER rule and evaluation model of perturbation analysis are constructed in “Construction of the models”. The real data of rolling bearing is analysed, and the validity of the proposed model is demonstrated in “Case study”. The conclusion is presented in “Conclusion”.

## Problem definition

Rolling bearings are easily affected by perturbations, resulting in performance fluctuations. The external perturbations include heavy load and high temperature, and the interior perturbations include wear, crack and seal damage. The adaptability of rolling bearing to different perturbations can be reflected by perturbation analysis. Therefore, a performance evaluation model is established based on the evaluation indicator system and the ER rule. Then, to further evaluate the reliability of rolling bearing under perturbations, a performance evaluation model considering perturbations is proposed. The main problems to be solved in this article are as follows:

### *Problem 1:*

***Construction of the evaluation indicator system***.

In practical engineering, vibration signals are easy to collect, so the diagnosis method based on vibration signals is generally adopted to evaluate the bearing performance. The characteristic indicators reflecting the bearing’s working conditions are extracted from vibration signals to evaluate the bearing’s performance. Common characteristic indicators are divided into time domain indicators and frequency domain indicators. Selecting appropriate indicators is the basis of evaluation. We established an evaluation indicator system:

1 $$ Indicator = \{ X_{1} ,X_{2} ,...,X_{i} ,...,X_{I} \} $$

where $Indicator$ is the indicator system, and $X_{i}$ is the indicator $i$. $I$ is the number of the different indicators.

### *Problem 2:*

***Construction of the rolling bearing performance evaluation model based on the ER rule***. There are many characteristic indicators that can reflect the performance of rolling bearing, such as the effective value, kurtosis, and centroid frequency. Among them, only RMS have national standards for reference. However, the effective value cannot completely evaluate the performance of rolling bearings. It needs to be combined with other characteristic indicators to obtain perfect evaluation results. How to effectively integrate multiple characteristics is the second problem to be solved. In view of this, we established an evaluation model based on the ER rule:

2 $$ z(t) = \Gamma [x(t),\omega ,r] $$

where $\Gamma ( \cdot )$ is a nonlinear function corresponding to the ER rule, $z(t)$ is the performance evaluation result at time $t$ without perturbation, $r$ is the indicator reliability, and $\omega$ is the indicator weight.

### *Problem 3:*

***Construction of the reliability evaluation model of rolling bearing performance considering perturbation***. During the operation of bearing, the performance reliability is closely related to internal and external interference factors. The influence of perturbation factors on each indicator should be fully considered. The third problem that needs to be solved is how to effectively characterize the influence of perturbations on evaluation indicators, and integrate them into the ER evaluation model to analyse the bearing’s performance reliability. In view of this, we established the evaluation model:

3 $$ Z(t) = \Phi [x(t),\omega^{^{\prime}} ,r^{^{\prime}} ,\sigma ,\Delta x(t)] $$

where $\Phi ( \cdot )$ is a nonlinear function corresponding to the model and $Z(t)$ is the reliability evaluation result under perturbation at time $t$. $x(t)$ is the indicator data at time $t$, $\omega^{^{\prime}}$ is the weight of the perturbation indicator, $r^{^{\prime}}$ is the reliability of the perturbation indicator, $\sigma$ is the perturbation intensity, and $\Delta x(t)$ is the perturbation variable at time $t$.

### *Problem 4:*

**Figure 1:**
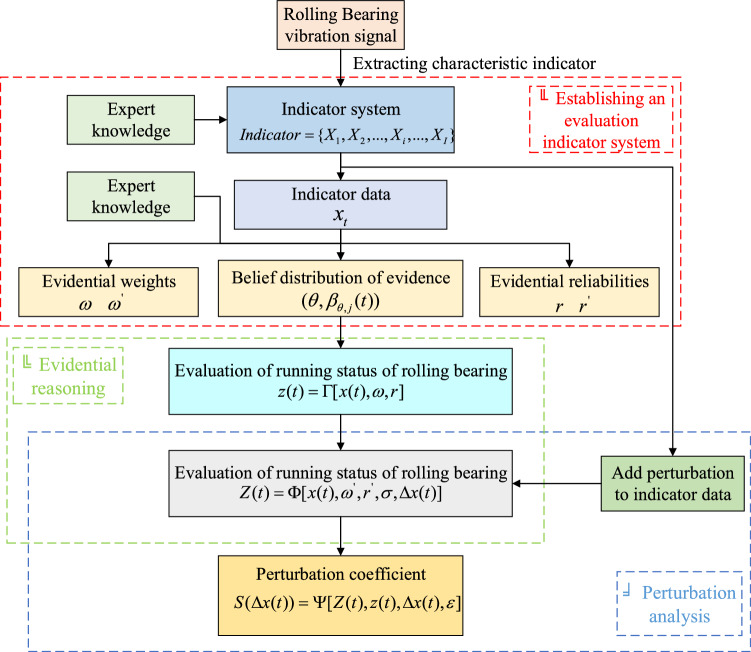
Evaluation of bearing performance considering perturbation.

***Construction of an adaptive quantization equation considering perturbation***. Rolling bearing has different adaptability to different interference factors. Determining the bearing’s adaptability to different perturbations and adjusting their working state is essential. We established a quantification equation of adaptability:

4 $$ S(\Delta x(t)) = \Psi [Z(t),z(t),\Delta x(t),\varepsilon ] $$

where $S(\Delta x(t))$ is the perturbation coefficient of the indicator data at time $t$ affected by the perturbation variable $\Delta x(t)$, and $\varepsilon$ is the maximum error. The perturbation coefficient can measure the bearing’s adaptability to different perturbations. If the perturbation influence on the performance of bearing exceeds a certain range, the running environment needs to be changed. The whole evaluation process is shown in Fig. 1, which consists of an evaluation indicator system, ER rule and perturbation analysis.

### *Remark 1:*

Traditional data-driven methods heavily rely on quantitative information, and it is difficult to use the subjective experience of decision makers. Knowledge-driven evaluation methods rely on expert experience, so it is difficult to obtain reasonable evaluation results. However, the ER rule can integrate quantitative information and qualitative knowledge to make reasonable evaluations and flexibly deal with uncertain information, and the reasoning process is interpretable. Therefore, it is reasonable to construct the state assessment model of a bearing with the ER rule.

## Construction of the models

First, the indicator evaluation system of rolling bearing is constructed. Second, the setting method of the evidence weight and the evidence reliability is determined, and the indicator information is converted into the form of a belief distribution. Third, the rolling bearing performance evaluation model based on the ER rule is described. Finally, a perturbation analysis model to quantify the bearing’s adaptability to perturbation is constructed in this section.

### Determine the evaluation indicator system

The time domain and frequency domain characteristics of vibration signals can show bearing’s performance, so different characteristic indicators are extracted^32^. Considering that there are errors in using a single indicator to evaluate performance, the combined analysis of multiple indicators can accurately reflect the bearing’s performance. The characteristic indicators are described as follows.

#### Effective value

The effective value, also known as the root mean square value (RMS), is used to reflect the signal energy. The higher the bearing wear degree is, the higher the effective value is. The equation for calculating the effective value is as follows:

5 $$ RMS = \sqrt {\frac{1}{{n_{s} }}\sum\limits_{i = 1}^{{n_{s} }} {y^{2} (i)} } $$

where $y(t)$ is a continuous vibration signal. $y(i)$ is a discrete-time vibration signal obtained by sampling and $i = 1,2,3,...,n_{s}$, $n_{s}$ is the number of vibration signals.

#### Peak value

It can reflect the impact force of a local fault point of bearing. The greater the impact is, the higher the peak value is. The peak value is often used to detect impact vibrations caused by cracks and spalling. It is calculated as follows:

6 $$ Y_{p} = \max (y(i)),i = 1,2,...,n_{s} $$

#### Peak factor

Considering that the effective value cannot clearly reflect discrete defects such as local peeling, scratching and notching on the bearing. although the total energy of the pulse waveform generated by such discrete defects is low, the peak degree of the waveform increases. It is appropriate to use the peak index to describe these defects. The calculation equation is as follows:

7 $$ C_{f} = Y_{p} /RMS = \max (y(i))/\sqrt {\frac{1}{{n_{s} }}\sum\limits_{i = 1}^{{n_{s} }} {y^{2} (i)} } $$

#### Average amplitude

The average amplitude can reflect the waveform index of rolling bearing’s vibration signal and can be calculated by

8 $$ S_{Y} = \sqrt {\frac{1}{{n_{s} }}\sum\limits_{i = 1}^{{n_{s} }} {y^{2} (i)} /\overline{y(t)} } $$

where $\overline{y(t)}$ is the average value of the continuous time vibration signal, and its calculation equation is as follows:

9 $$ \overline{y(t)} = \frac{1}{{n_{s} }}\sum\limits_{i = 1}^{{n_{s} }} {y(i)} $$

#### Kurtosis

Kurtosis is sensitive to impact signals and is suitable for the diagnosis of surface damage faults. Kurtosis is a fourth-order statistic. It is difficult to distinguish the fault signal from the noise, and kurtosis addresses the fourth power of amplitude. High amplitude is prominent, and low amplitude is suppressed, so that the pulse reflecting the fault characteristic information can be extracted from the pulse modulation signal mixed with noise. The kurtosis is calculated as follows:

10 $$ Kurtosis\{ y(t)\} = \frac{{\frac{1}{{n_{s} }}\sum\nolimits_{i = 1}^{{n_{s} }} {(y(i) - \overline{y(t)} )^{4} } }}{{\left( {\frac{1}{{n_{s} }}\sum\nolimits_{i = 1}^{{n_{s} }} {(y(i) - \overline{y(t)} )^{2} } } \right)^{2} }} $$

#### Centroid frequency

The centroid frequency (CF) can describe the signal frequency in the spectrum, and reflect the distribution of the signal power spectrum. It is the weighted average of the amplitude of the power spectrum and can be calculated by

11 $$ CF = \frac{{\sum\nolimits_{{f = f_{1} }}^{{f_{2} }} {f*P(f)} }}{{\sum\nolimits_{{f_{1} }}^{{f_{2} }} {P(f)} }} $$

where $f_{1}$ and $f_{2}$ are the frequency domains, and $P(f)$ is the power spectrum of the signal obtained by the Fourier transform which can be calculated by

12 $$ P(f) = \sum\limits_{n = 1}^{N} {x(n)e^{ - j\omega n} } $$

where $x(n)$ is a discrete signal series, $n$ is the discrete signal sampling point. $j$ is the imaginary unit of the complex number, and $\omega$ is the angular frequency.

#### Root mean square frequency

The root mean square frequency (RMSF) is the arithmetic square root of the mean square frequency (MSF), and it is the weighted average of the square of the signal frequency. The RMSF is calculated as follows:

13 $$ RMSF = \sqrt {\frac{{\sum\nolimits_{{f = f_{1} }}^{{f_{2} }} {f^{2} *P(f)} }}{{\sum\nolimits_{{f_{1} }}^{{f_{2} }} {P(f)} }}} $$

Considering the large difference in the dynamic range of different characteristic indicators, the maximum and minimum values may differ by many orders of magnitude, so the data of each indicator are converted into decibels (dB) to highlight the change.

### Determining evaluation indicator reliability and weight

The ER rule is used to build the performance evaluation model of rolling bearing. Each characteristic indicator is regarded as a piece of evidence, and each piece of evidence has two attributes of reliability and weight^33^. The distance-based method and coefficient of variation (COV) method are used to calculate the reliability and weight of evidence, respectively^26^.

#### Approach for calculating the indicator reliability

The vibration signal data of the rolling bearing are monitored by sensors. However, the detection data may fluctuate greatly due to the influence of noise. Therefore, the reliability must be considered when fusing the indicator data. Suppose there are $n$ indicators, and the reliability of indicators is $r_{1} ,r_{2} ,...,r_{n}$. Obtained from the distance-based method:

14 $$ d_{i,k} (x_{i} (k),\overline{{x_{i} }} ) = |x_{i} (k) - \overline{{x_{i} }} |,k = 1,2,...,K $$

where $x_{i} (k)$ is the monitoring data of indicator $X_{i}$ at time $k$, $\overline{{x_{i} }}$ is the average value of all indicator data in time period $K$, and $\overline{{x_{i} }} = \frac{1}{K}\sum\nolimits_{k = 1}^{K} {x_{i} (k)}$. $d_{i,k} (x_{i} (k),\overline{{x_{i} }} )$ is the distance between $x_{i} (k)$ and $\overline{{x_{i} }}$. Then the average distance of all test data in $K$ time periods is as follows:

15 $$ \overline{{D_{i} }} = \frac{1}{K}\sum\limits_{k = 1}^{K} {d_{i,k} } = \frac{1}{K}\sum\limits_{k = 1}^{K} {\left| {x_{i} (k) - \overline{{x_{i} }} } \right|} $$

The reliability of the indicator is defined as follows:

16 $$ r_{i} = \frac{{\overline{{D_{i} }} }}{{\max (d_{i,k} (x_{i} (k),\overline{{x_{i} }} ))}} $$

where $\max (d_{i,k} (x_{i} (k),\overline{{x_{i} }} ))$ is the maximum value of $d_{i,k} (x_{i} (k),\overline{{x_{i} }} )$,and $d_{i,k} (x_{i} (k),\overline{{x_{i} }} )$ can characterize the fluctuations of each indicator. According to the analysis of bearing’s running characteristics, the larger the fluctuation is, the more unreliable the indicator data are. Therefore, the determination of reliability $r_{i}$ is reasonable.

#### Approach for calculating indicator weighting

The indicator weight represents the relative importance of characteristic indicators in the evaluation system. The COV method determines the indicator weight by analysing the fluctuation degree of different indicators. Suppose there are $n$ indicators, and the weight of indicators is $\omega_{1} ,\omega_{2} ,...,\omega_{n}$. Obtained from the COV method:

17 $$ v_{i} = \frac{{\sqrt {\frac{1}{K - 1}\sum\nolimits_{k = 1}^{K} {(x_{i} (k) - \overline{{x_{i} }} )^{2} } } }}{{\overline{{x_{i} }} }} $$

where $v_{i}$ is the standard deviation of the evaluation indicator $i$ and $\overline{{x_{i} }}$ is the average value of all indicator data in time period $K$. Then, the variation coefficient is normalized, and the weight of evaluation indicator $i$ is calculated by

18 $$ \omega_{i} = \frac{{v_{i} }}{{\sum\nolimits_{j = 1}^{n} {v_{j} } }} $$

where $v_{i}$ is fluctuation degree of the evaluation indicator. The fluctuation of the indicator data represents the ability of indicator to respond to the abnormal data. The bigger the indicator volatility is, the higher the weight is.

##### *Remark 2:*

Reliability is signal-to-noise ratio, namely the proportion of noise in the information, which is assumed not to seriously affect the original information. Weight represents the sensitivity of information to system characteristics, and this information is the ideal information without noise.

#### Indicator data standardization

According to the analysis of the historical engineering and the understanding of the working principle, the indicator reference grades and reference values are determined. Then, based on the reference grades and reference values, the rule-based information transformation method^34^ is adopted to transform the indicator data into the form of a belief distribution.

19 $$ \left\{ \begin{gathered} p_{i,j} = \frac{{h_{i,j + 1} - x_{i,j} }}{{h_{i,j + 1} - h_{i,j} }},h_{i,j} \le x_{i,j} \le h_{i,j + 1} ,j = 1,...,J - 1 \hfill \\ \hfill \\ p_{i,j + 1} = 1 - p_{i,j} ,h_{i,j} \le x_{i,j} \le h_{i,j + 1} ,j = 1,...,J - 1 \hfill \\ \hfill \\ p_{i,k} = 0,k = 1,...,J;k \ne j,j + 1 \hfill \\ \end{gathered} \right. $$

where $h_{i,j} (i = 1,2,...,I;j = 1,2,...,J)$ is the reference value of the indicator $X_{i}$, $x_{i,j}$ is the input data of indicator $X_{i}$, $J$ is the number of reference values and $h_{i,j + 1} \ge h_{i,j}$.

##### *Remark 3:*

Before the evaluation, we interpret the sample data to judge whether the data are within a reasonable range. The unreasonable data are supposed to be replaced with new data. The reference values are supposed to meet the fluctuation range of the sample data in the setting process, and avoid the sample data exceeding the range of reference values.

### Evaluation model of rolling bearing performance based on the ER rule

After determining the reliability and weight of evidence and standardizing the indicator data, the ER rule is used to fuse the information of characteristic indicators and evidence parameters. The bearing’s performance is evaluated in the form of a belief distribution and expected utility. Suppose that a node collects $T$ pieces of data, and each piece of information has $I$ indicators. The input indicator data are $x_{i} (i = 1, \ldots ,I)$, which is represented as evidence $e_{i} (i = 1, \ldots ,I)$.The frame of discernment is composed of $N$ evaluation levels $H_{n} (n = 1, \ldots ,N)$, namely $\Theta = \{ H_{1} , \ldots ,H_{N} \}$. After data standardization, the evidence can be expressed as the following form of belief distribution:

20 $$ e_{i} = \{ (H_{n} ,p_{n,i} ),n = 1, \ldots ,N;(\Theta ,p_{\Theta ,i} )\} $$

where $p_{n,i}$ is the belief degree of the evaluation scheme evaluated as evaluation level $H_{n}$ under evidence $e_{i}$, and $\Theta$ is the discernment framework including all evaluation levels. $p_{\Theta ,i}$ is the belief degree of indicator $i$ relative to discernment framework $\Theta$, namely global ignorance, and $p_{\Theta ,i}$ satisfies $0 \le p_{n,i} \le 1$, $\sum\nolimits_{n = 1}^{N} {p_{n,i} \le 1}$. The evidence reliability is $r_{i} (i = 1, \ldots ,I)$, which satisfies ${0} \le r_{i} \le 1$. The evidence weight is $\omega_{i} (i = 1, \ldots ,I)$, which satisfies $0 \le \omega_{i} \le 1$ after normalization. The weighted belief distribution of the evidence $i$ with reliability is:

21 $$ m_{i} = \{ (H_{n} ,\tilde{m}_{n,i} ),\forall \theta \subseteq \Theta ;(p(\Theta ),\tilde{m}_{p(\Theta ),i} )\} $$

where $P(\Theta )$ is a power set and $\tilde{m}_{n,i}$ is the mixed probability quality of indicator $i$ under level $H_{n}$ and satisfies:

22 $$ \tilde{m}_{n,i} = \left\{ \begin{gathered} 0, \, H_{n} = \emptyset \hfill \\ c_{rw,i} m_{n,i} , \, H_{n} \subseteq \Theta ,H_{n} \ne \emptyset \hfill \\ c_{rw,i} (1 - r_{i} ), \, H_{n} = P(\Theta ) \hfill \\ \end{gathered} \right. $$

23 $$ \sum\limits_{n = 1}^{N} {\tilde{m}_{n,i} + \tilde{m}_{P(\Theta ),i} = 1} $$

where $c_{rw,i} = {1 \mathord{\left/ {\vphantom {1 {(1 + \omega_{i} }}} \right. \kern-\nulldelimiterspace} {(1 + \omega_{i} }} - r_{i} )$ is the regularization coefficient. $m_{n,i}$ is the basic probability quality of indicator $i$ under level $H_{n}$, $\emptyset$ is an empty set, and $m_{n,i} = \omega_{i} p_{n,i}$.

Each indicator can describe the characteristics of rolling bearing vibration signals from different angles. The data of each indicator are standardized and processed by Eq. (19), and its belief distribution is expressed by Eq. (20). For any two indicators $e_{i}$ and $e_{j}$, if their belief distribution is expressed by Eq. (20), then their joint support belief degree $p_{n,e(2)}$ for the evaluation grade $H_{n}$ is:

24 $$ \begin{aligned} \hat{m}_{n,e(2)} = & \left[ {(1 - r_{i} )m_{n,j} + (1 - r_{j} )m_{n,i} } \right] \\ & \; + \sum\nolimits_{\begin{subarray}{l} A \cap B = H_{n} \\ A,B \subseteq \Theta \end{subarray} } {m_{A,i} m_{B,j} } \\ \end{aligned} $$

25 $$ p_{n,e(2)} = \left\{ \begin{gathered} 0, \, H_{n} = \emptyset \hfill \\ \frac{{\hat{m}_{n,e(2)} }}{{\sum\nolimits_{D \subseteq \Theta } {\hat{m}_{D,e(2)} } }} \hfill \\ \end{gathered} \right.,\quad H_{n} \subseteq \Theta ,\;H_{n} \ne \emptyset $$

$\hat{m}_{n,e(2)}$ is the unnormalized combination probability quality assigned to the evaluation grade $H_{n}$ after combining $e_{i}$ and $e_{j}$. $\hat{m}_{D,e(2)}$ is the unnormalized combination probability quality assigned to the evaluation grade $D$ after combining $e_{i}$ and $e_{j}$. $A$, $B$ and $D$ represent subsets of the whole set.

The combined belief $p_{n,e(I)}$ of $H_{n}$ is determined by the following formula for $I$ pieces of evidence:

26 $$ \begin{aligned} \hat{m}_{n,e(k)} = & \left[ {(1 - r_{k} )m_{n,e(k - 1)} + m_{p(\Theta ),e(k - 1)} m_{n,k} } \right] \\ & + \sum\nolimits_{{A \cap B = H_{n} }} {m_{A,e(k - 1)} m_{B,k} } \\ \end{aligned} $$

27 $$ \hat{m}_{p(\Theta ),e(k)} = (1 - r_{k} )m_{p(\Theta ),e(k - 1)} $$

28 $$ m_{n,e(k)} = \left\{ \begin{gathered} 0, \, H_{n} = \emptyset \hfill \\ \frac{{\hat{m}_{n,e(k)} }}{{\sum\nolimits_{D \subseteq \Theta } {\hat{m}_{D,e(k)} + \hat{m}_{P(\Theta ),e(k)} } }},H_{n} \ne \emptyset \hfill \\ \end{gathered} \right. $$

29 $$ p_{n,e(k)} = \left\{ \begin{gathered} 0, \, H_{n} = \emptyset \hfill \\ \frac{{\hat{m}_{n,e(k)} }}{{\sum\nolimits_{D \subseteq \Theta } {\hat{m}_{D,e(k)} } }} \hfill \\ \end{gathered} \right.,H_{n} \subseteq \Theta ,H_{n} \ne \emptyset $$

Among them, $k = 3,4, \ldots ,I$, $m_{n,e(k - 1)}$ and $m_{A,e(k - 1)}$ are the normalized combination probability qualities assigned to grade $H_{n}$ and grade $A$ after the combination of the first $k - 1$ indicators. $\hat{m}_{p(\Theta ),e(k)}$ is the unnormalized probability mass assigned to the power set after the fusion of the first $k$ indicators, and $m_{p(\Theta ),e(k - 1)}$ is the normalized probability mass assigned to the power set after the fusion of the first $k - 1$ indicators. $\hat{m}_{n,e(k)}$ and $\hat{m}_{D,e(k)}$ are the unnormalized combination probability qualities assigned to grades $H_{n}$ and $D$ after the combination of the first $k$ indicators, respectively. $p_{n,e(k)}$ is the belief degree of the first $k$ indicators to the evaluation level $H_{n}$ after fusion which meets the requirements of $m_{n,e(1)} = m_{n,1}$ and $m_{p(\Theta ),e(1)} = m_{p(\Theta ),1}$. Through the above iterative algorithm, the comprehensive evaluation results are obtained:

30 $$ e(I) = \{ (H_{n} ,p_{n,e(I)} ),\;\;n = 1, \ldots ,N,(\Theta ,p_{\Theta ,e(I)} )\} $$

Suppose the utility of the reference level $H_{n}$ is $u(H_{n} )$. The expected utility of the evaluation results can be obtained according to the utility calculation method proposed in reference^32^:

31 $$ u(e(I)) = \sum\limits_{n = 1}^{N} {u(H_{n} )p_{n,e(I)} + u(\Theta )p_{\Theta ,e(I)} } $$

where $u$ is the expected utility of the evaluation, which can be used to evaluate the performance of the rolling bearing. Equation (31) is $\Gamma ( \cdot )$ as shown in Eq. (2).

### Performance reliability evaluation model of rolling bearing considering perturbation

**Figure 2:**
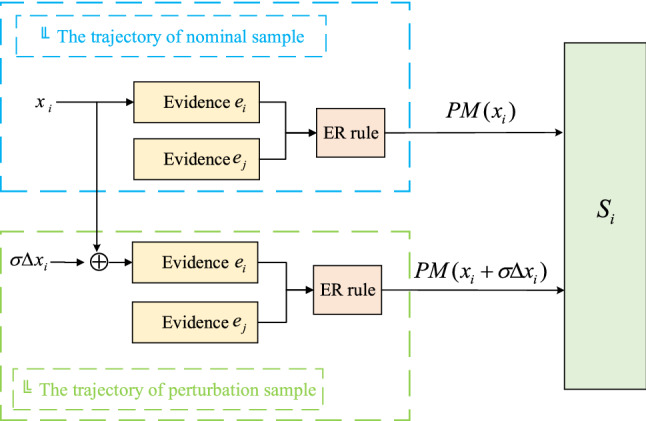
Rolling bearing performance reliability model considering perturbation.

In practical engineering, rolling bearing will be affected by sealing damage, heavy loads, cracks and other interference factors, resulting in reliability reduction. To further analyse the rolling bearing’s adaptability to interference factors during operation, the perturbation analysis method is used to simulate interference environment, as shown in Fig. 2.

As shown in Fig. 2, the upper part is a nominal sample trajectory of performance evaluation, corresponding to two pieces of evidence $e_{i}$ and $e_{j}$, $x_{i}$ is the input parameter, and the output utility $PM(x_{i} )$ is generated under the combination of the ER rule, namely, the performance evaluation results. The lower part is a trajectory of the perturbation sample. Based on the nominal trajectory, the input parameter of evidence $e_{i}$ is changed to $x_{i} + \sigma \Delta x_{i}$; then, the output utility $PM(x_{i} + \sigma \Delta x_{i} )$ of the two pieces of evidence is generated under the combination of the ER rule, namely, the performance reliability evaluation result considering the perturbation. $PM(x_{i} )$ and $PM(x_{i} + \sigma \Delta x_{i} )$ are the same as the expected utility $u$ in Eq. (31).

Suppose the identification framework of the evaluation model is $\Theta = \{ H_{1} , \ldots ,H_{N} \}$ and the evaluation indicator is $e_{i} (i = 1, \ldots ,I)$, the corresponding reliability and weight of each evidence are $\{ r^{^{\prime}}_{1} ,r^{^{\prime}}_{2} , \ldots ,r^{^{\prime}}_{I} \}$ and $\{ \omega^{^{\prime}}_{1} ,\omega^{^{\prime}}_{2} ,...,\omega^{^{\prime}}_{I} \}$ respectively, and the reference value is $h_{i,j} (i = 1,2, \ldots ,I;j = 1,2, \ldots ,J)$. The belief distribution of indicator evidence $e_{i}$ under perturbation condition is calculated by Eq. (19):

32 $$ e_{i} = \left\{ {\left( {H_{l} ,\frac{{x_{i} + \sigma_{i} \Delta x_{i} - h_{l + 1} }}{{h_{l} - h_{l + 1} }}} \right),\left( {H_{l + 1} ,\frac{{h_{l} - x_{i} - \sigma_{i} \Delta x_{i} }}{{h_{l} - h_{l + 1} }}} \right),(H_{k} ,0)} \right\} $$

where $k \in [1,N]$, $k \ne l,l + 1$, $h_{1}$ and $h_{I}$ correspond to the maximum and minimum reference values respectively, $h_{l + 1} \le x_{i} \le h_{l}$. The rule-based information transformation method is used to transform it into the belief distribution form, the ER rule is used to fuse all indicators, and then the bearing’s performance reliability evaluation results can be obtained. To quantify the adaptability of different perturbations, the perturbation coefficient is proposed as follows:

33 $$ S_{i} = \frac{{PM(x_{i} + \sigma \Delta x_{i} ) - PM(x_{i} )}}{{\Delta x_{i} }} $$

If $S_{i} \le |\varepsilon |$, $\varepsilon$ is the maximum error of the perturbation coefficient, it can be considered that the influence of perturbation on the performance reliability of rolling bearing is acceptable. Otherwise, the rolling bearing must be changed or adjusted.

### Inference process of the performance reliability of rolling bearing considering perturbation

The bearing’s performance model and performance reliability model are established in  “Evaluation model of rolling bearing performance based on the ER rule” and “Performance reliability evaluation model of rolling bearing considering perturbation”, respectively. In this section, a general method of the model is analysed.

**Figure 3:**
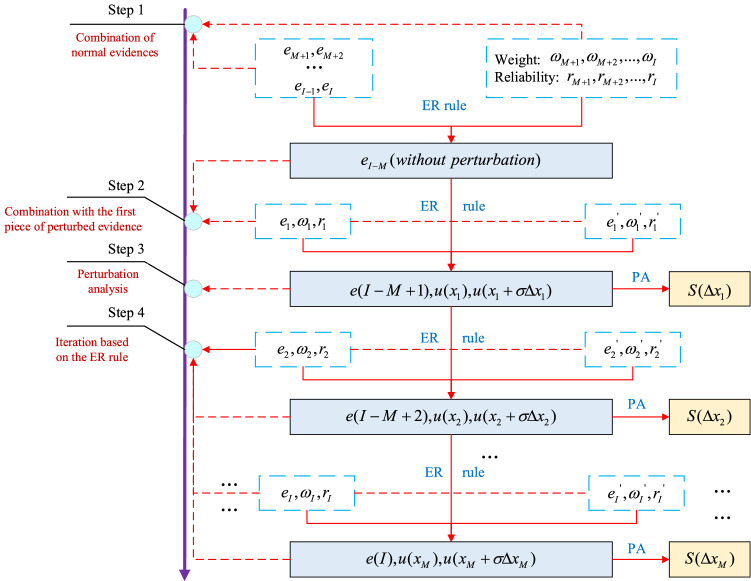
The overall process of ER rule-PA.

Suppose there are $I$ pieces of independent evidence $\{ e_{1} ,e_{2} , \ldots ,e_{I} \}$ and $M(M \le I)$ pieces of independent evidence. Since it has been demonstrated that the fusion results by the ER rule cannot be influenced by the order of evidence combination^21^, $I$ pieces of evidence are reordered as $\{ e_{1} , \ldots ,e_{i} , \ldots ,e_{M} ,e_{M + 1} , \ldots ,e_{j} , \ldots ,e_{I} \}$. Based on this feature, the implementation of ER rule-PA is divided into four steps, as shown in Fig. 3. The generalized method of ER rule-PA is concluded as follows:

*Step 1*: Combine $(I - M)$ pieces of normal evidence based on the ER rule as Eqs. (19)–(31), and $e(I - M)$ is calculated by Eq. (30).

*Step 2*: Combine the previously combined assessment result $e(I - M)$ with the first piece of perturbed evidence based on the ER rule to obtain $e(I - M + 1)$. Then, the expected utility $u(x_{1} + \sigma \Delta x_{1} )$ under perturbation and the expected utility $u(x_{1} )$ without perturbation are calculated by Eq. (31).

*Step 3*: Perform a perturbation analysis on the result of step 2, and the perturbation coefficient $S(\Delta x_{1} )$ is calculated by Eq. (33).

*Step 4*: Combine $e(I - M + 1)$ in step 2 with the remaining $(M - 1)$ pieces of evidence by recursively applying the ER rule as step 1. Then, perform a perturbation analysis as step 3 successively.

#### *Remark 4:*

The offline data of rolling bearing vibration signals are used to construct the ER evaluation model, which can monitor the bearing system in real time. We replace the overall time period $K$ in Eqs. (14)–(18) with its sub-time period $k_{i}$ and then iteratively calculate the reliabilities and weights in real time to achieve online evaluation.

## Case study

To demonstrate the validity of the established model, the performance and performance reliability of rolling bearing are evaluated based on the indicator evaluation system established in “Determine the evaluation indicator system”. The performance evaluation model established in “Problem definition”. The heavy load strength and sealing of rolling bearing are taken as the sources of perturbation. Different characteristic indicators are selected for evaluation in “Study background” and “Experiment 1”.

### Study background

The experimental data are a rolling bearing life-cycle public data set prepared by the NSF I/UCR Intelligent Maintenance System Center of the University of Cincinnati. In the test platform, the AC motor rotates at a constant speed of 2000 RPM and is connected to the shaft through a friction belt. The vibration signals are collected at an interval of 10 min for one second, and the number of points is 20,480. The sampling frequency of signals is 20 kHz. In this article, the data collected in channel 1 are selected as experimental data. Due to the aggravation of rolling bearing wear in the later period, the data have become invalid, so the first 948 groups of 982 vibration signals are selected as experimental data, and the test time is 158 h.

### Experiment 1

#### Indicator data analysis

**Figure 4:**
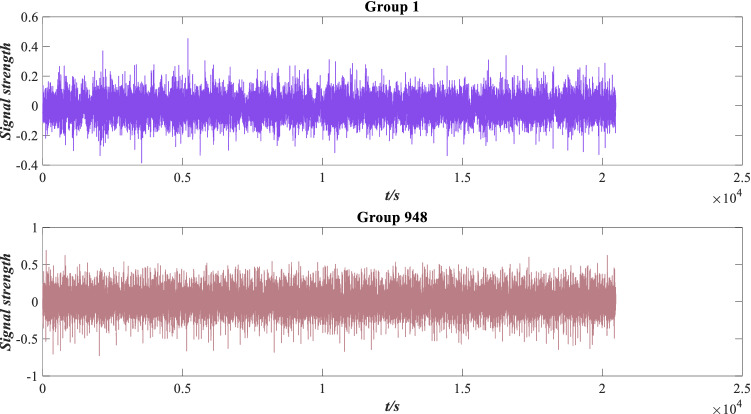
Vibration signal distribution.

Bearing’s vibration signal should fluctuate steadily within a small range. If the fluctuation range exceeds a specific threshold value, it indicates that the performance of the rolling bearing deteriorates and the probability of failure increases. The distribution of vibration signals is shown in Fig. 4. The group 1 and group 984 vibration signals are selected for display.

**Figure 5:**
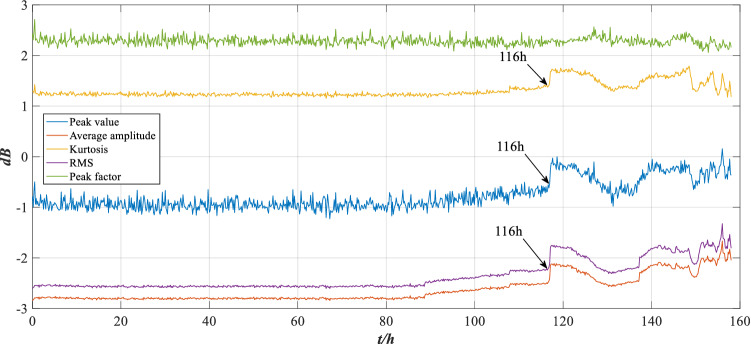
The trend of characteristic indicators.

As shown in Fig. 4, the vibration signal fluctuation of group 948 is significantly higher than that of group 1, indicating that the wear is aggravated. To describe the changing state of the vibration signal, the RMS, peak value, average amplitude, peak factor and kurtosis feature are extracted and analysed, as shown in Fig. 5.

As shown in Fig. 5, the RMS, peak value, kurtosis and average amplitude have obvious variation trends. The performance of the rolling bearing is good for the first 80 h, but the bearing wears slightly after 80 h, the wear becomes worse after 116 h, and the characteristic indicators show an irregular change trend. Three characteristic indicators of RMS, peak value and kurtosis are selected for analysis in this section.

#### Performance evaluation of rolling bearing based on the ER rule

The evaluation model proposed “Problem definition” is used to evaluate the rolling bearing’s performance, and the rule-based input information transformation method is adopted to unify the data of each indicator into the form of a belief distribution. The evaluation level is set as high, medium and low, namely, the identification frame is $\Theta = \{ (H_{1} ,high),(H_{2} ,medium),(H_{3} ,low)\}$. According to expert knowledge, the reference level and reference value of each evaluation indicator are divided as shown in Table 1. The “high” and “low” reference values of RMS, peak value and kurtosis are the minimum value and maximum value of indicator data, respectively, and since the bearing wear is aggravated at 80–116 h, the “medium” reference value is the denser point of data distribution between 80 and 116 h.

**Table 1:** Indicator evaluation level and reference value.

	$H_{1}$	$H_{2}$	$H_{3}$
RMS	− 2.604	− 2.234	− 1.325
Peak value	− 1.228	− 0.622	0.156
Kurtosis	1.151	1.368	1.789

In the performance evaluation model, the indicator data are normalized and transformed by the rule-based method according to Eq. (19), and the belief distribution results shown in Eq. (20) are obtained. The following is an example of a belief conversion: suppose the kurtosis indicator of vibration signal is 1.30 at time $t$, then $p_{1,2} (t) = \frac{1.30 - 1.151}{{1.368 - 1.151}} = 0.687$, $p_{1,1} (t) = 1 - \frac{1.30 - 1.151}{{1.368 - 1.151}} = 0.313$, $p_{1,3} (t) = 0$. The belief distribution form of this indicator can be expressed as:

34 $$ e_{1} (t) = \{ (H_{1} ,0.313),(H_{2} ,0.687),(H_{3} ,0)\} $$

**Figure 6:**
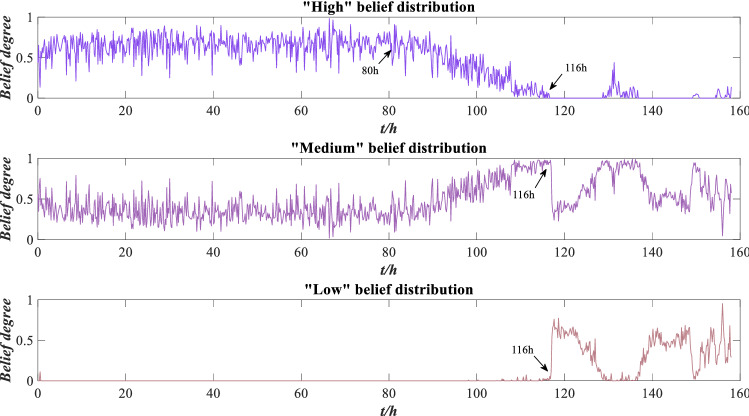
Performance evaluation results of rolling bearing.

The data of other indicators are also converted by this method. Suppose the reliability of the RMS, peak value and kurtosis are $r_{1}$, $r_{2}$ and $r_{3}$ respectively, and the weights are $\omega_{1}$, $\omega_{2}$ and $\omega_{3}$, respectively. The distance-based method is used to calculate the indicator reliability according to Eqs. (14)–(16), which are $r_{1} = 0.2254$, $r_{2} = 0.3012$ and $r_{3} = 0.2414$, respectively. The COV method is used to calculate indicator weights according to Eqs. (17)–(18), which are $\omega_{1} = 0.1992$, $\omega_{2} = 0.6135$ and $\omega_{3} = 0.1873$, respectively. The ER rule is used to fuse the above indicator information according to Eqs. (19)–(30), and the evaluation results of rolling bearing performance are obtained as shown in Fig. 6.

As shown in Fig. 6, the performance evaluation results are mostly concentrated around the “high” belief degree, followed by the “medium” belief degree, and a few evaluation results are in the “low” belief degree, mainly distributed after 116 h, which conforms to the real operation situation of rolling bearing. Before 116 h, the bearing’s performance is good, and after 116 h, the bearing wear is serious, resulting in poor performance.

**Figure 7:**
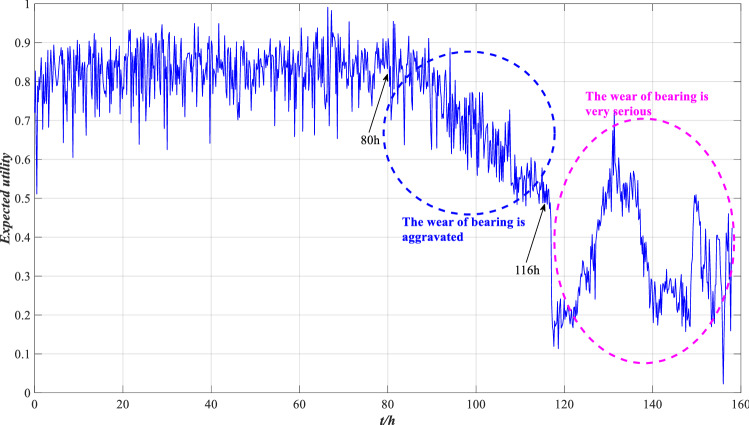
Expected utility of rolling bearing performance.

Suppose the utility of the reference grade is $u(H_{1} ) = 1$, $u(H_{2} ) = 0.5$ and $u(H_{3} ) = 0$, and the expected utility of performance can be calculated by Eq. (31), as shown in Fig. 7.

**Figure 8:**
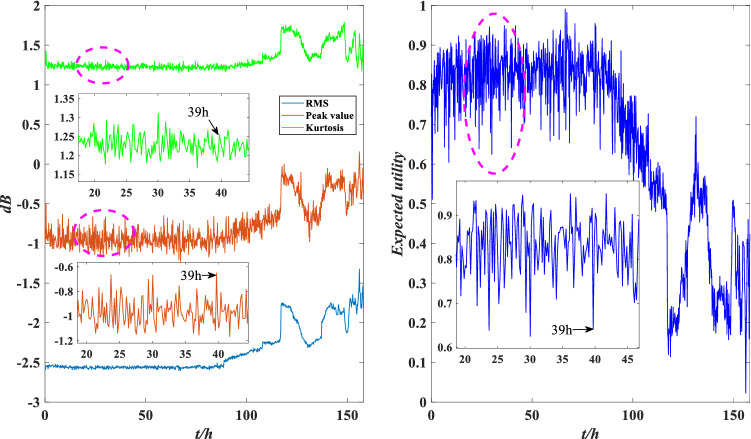
Local indicators and utility comparisons.

**Figure 9:**
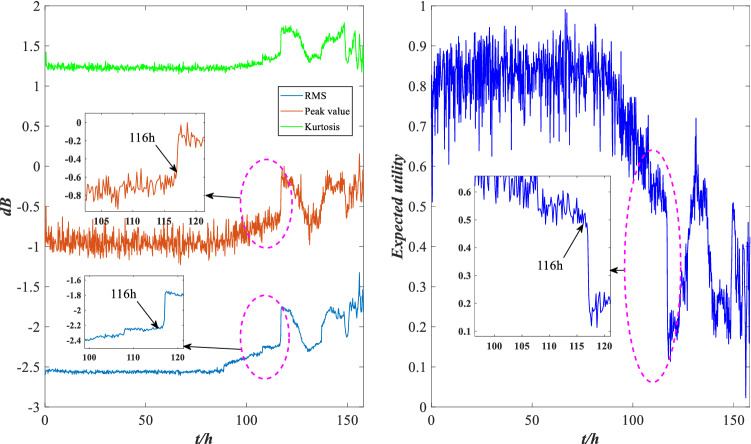
Local indicators and utility comparisons.

**Figure 10:**
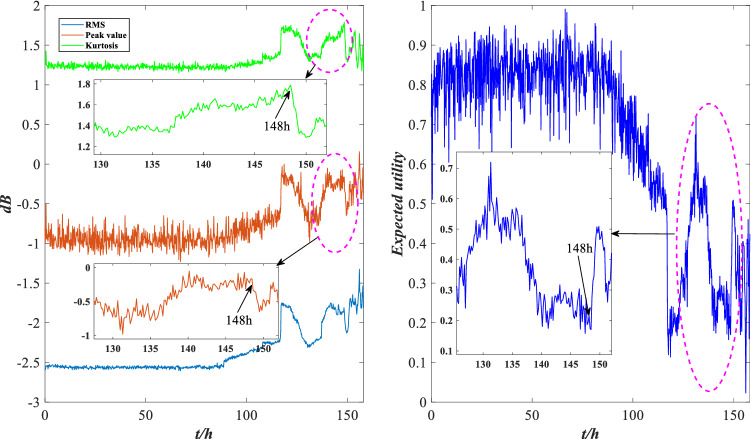
Local indicators and utility comparisons.

According to Fig. 7, the expected utility of bearing is distributed between 0.5 and 1 before 116 h, and the wear is aggravated between 80 and 116 h, but the overall performance is good. After 116 h, the expected utility drops sharply and fluctuates sharply between 120 and 158 h, with the overall distribution below 0.5, indicating that bearing wear is serious after 116 h and the performance is unreliable. This is consistent with the results shown in Fig. 6, indicating that the evaluation model is effective. To further demonstrate the effectiveness of this method, local images with drastic changes in utility are selected for comparison with Fig. 5, as shown in Figs. 8, 9 and 10.

The comparison between the indicator data and the utility of the rolling bearing under normal operation is shown in Fig. 8. The peak value and kurtosis value rise slightly at 39 h, and the utility shows a downtrend, indicating that the performance is reduced, but it still fluctuates within a normal range. The comparison between the indicator data and the utility of the abnormal operation is shown in Figs. 9 and 10. The RMS and peak value increase sharply at 116 h, indicating that the bearing’s performance decreases sharply. The peak value and kurtosis value decreased slightly at 148 h, indicating that the performance improved slightly, and the utility also shows an uptrend at 148 h. Therefore, the evaluation results of the model are consistent with the actual situation.

#### Performance reliability analysis of rolling bearing considering perturbation

Bearings will be affected by various disturbance factors, so the reliability evaluation model considering perturbation is used to evaluate the performance reliability. Four types of perturbation environments are simulated, namely, low load and high sealing, low load and low sealing, high load and high sealing, high load and low sealing. The corresponding perturbation intensities are 0.0015, 0.0030, 0.0045 and 0.0060, respectively. The perturbation variable represents the variable of the actual indicator data in operation with respect to the perceived information without perturbation. It has the following characteristics:

1. The generation of perturbation is irregular and random.
2. The generation of perturbation variables accords with the characteristics of normal distribution.

**Figure 11:**
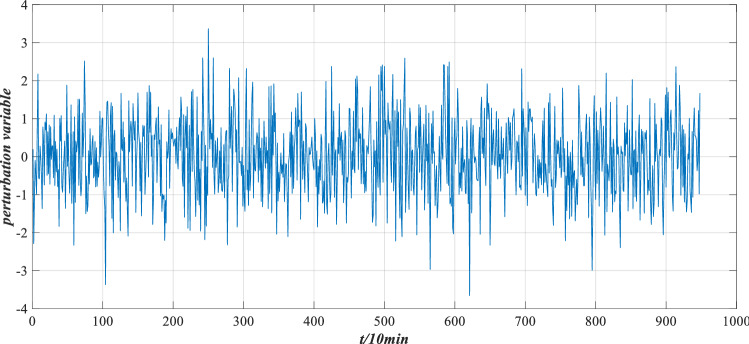
Perturbation variable distribution.

**Figure 12:**
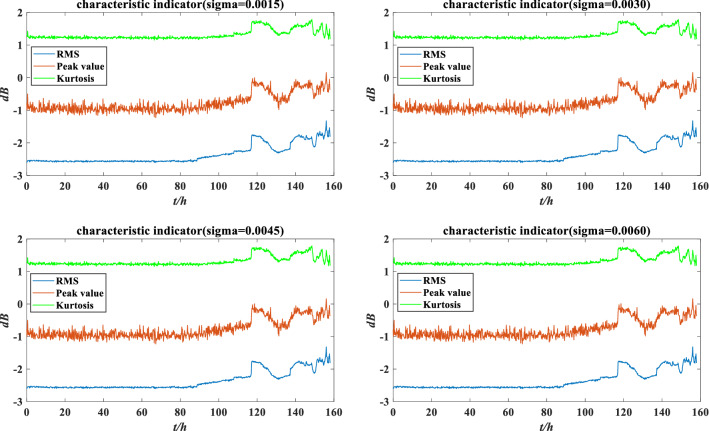
The characteristic indicators under different perturbation intensities.

The distribution of perturbation variable $\Delta x$ is shown in Fig. 11. After the addition of perturbation, the data of each characteristic indicator changed. The distribution of indicator data under different perturbation intensities is listed in Fig. 12.

At present, the perturbation intensity mainly depends on the expert knowledge. More scientific calculation methods will be used in the future to improve the rationality of perturbation intensity. Since the rolling bearing is affected by perturbation, each indicator should be recalculated. In addition, the stability of each indicator in the disturbance environment is different, and the reliability and weight of each indicator also change. The reliability and weight of each indicator under different perturbation intensities are calculated according to Eqs. (14)–(18), as shown in Tables 2 and 3.

**Table 2:** Indicator reliability under different perturbation intensities.

$r$	$\sigma = 0.0015$	$\sigma = 0.0030$	$\sigma = 0.0045$	$\sigma = 0.0060$
RMS	0.2251	0.2247	0.2244	0.2241
Peak value	0.3016	0.3027	0.3038	0.3050
Kurtosis	0.2446	0.2466	0.2488	0.2513

**Table 3:** Indicator weight under different perturbation intensities.

$\omega$	$\sigma = 0.0015$	$\sigma = 0.0030$	$\sigma = 0.0045$	$\sigma = 0.0060$
RMS	0.1991	0.1991	0.1990	0.1988
Peak value	0.6134	0.6131	0.6128	0.6123
Kurtosis	0.1875	0.1878	0.1882	0.1889

**Figure 13:**
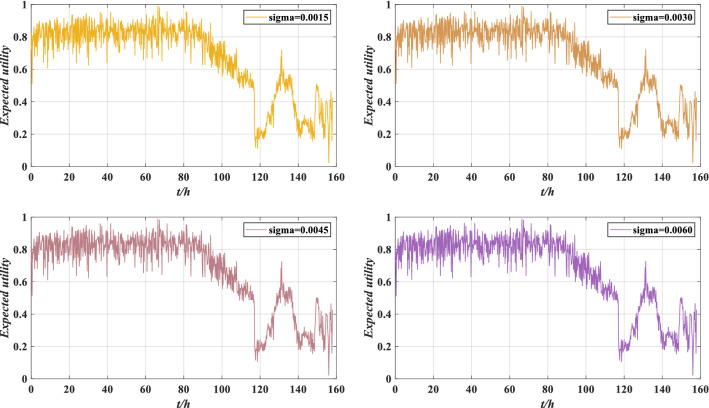
Expected utility of rolling bearing performance under different perturbation intensities.

Through the ER rule fusion and utility calculation, the expected utility under perturbation is obtained, as shown in Fig. 13.

According to Fig. 13, there are slight deviations in the evaluation results under different perturbations, but the overall evaluation result is similar to the utility without perturbation. The model can still accurately evaluate the performance under perturbations, which has a certain robustness. However, considering that different perturbation intensities will lead to different evaluation results, it is essential to verify the performance reliability of rolling bearing under different perturbation intensities.

**Figure 14:**
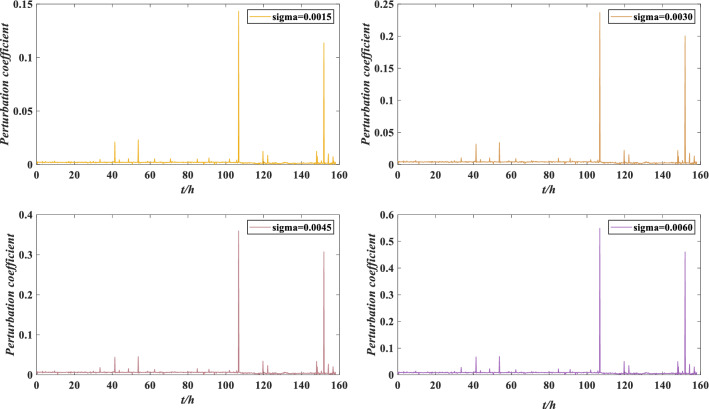
Perturbation coefficients under different perturbation intensities.

The perturbation coefficients under different perturbation intensities are calculated by Eq. (33) and their absolute values are shown in Fig. 14. The perturbation coefficient refers to the change in the expected utility under perturbation compared with the expected utility without perturbation, which is used to measure the performance reliability of rolling bearing against different perturbation conditions. The smaller the perturbation coefficient is, the stronger the ability to resist the perturbation condition. Otherwise, the bearing is damaged and must to be repaired or replaced.

**Figure 15:**
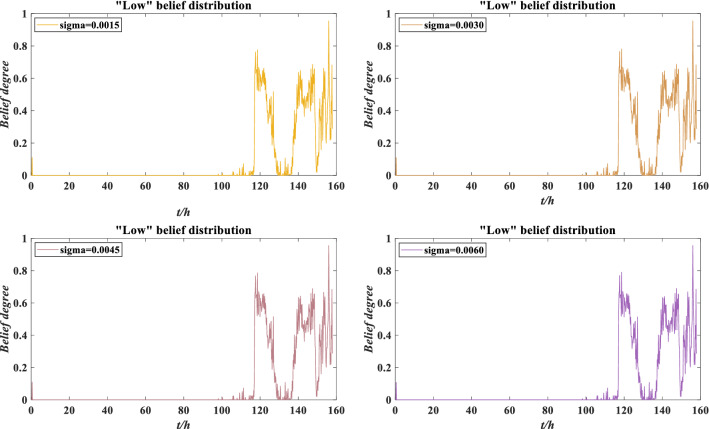
Low belief degree distribution of performance evaluation results under different perturbation intensities.

According to Fig. 14, the absolute value of the perturbation coefficient increases with increasing perturbation intensity, which is consistent with the negative impact of perturbation on rolling bearing. Moreover, the belief distribution of rolling bearing performance grade “low” under different perturbation intensities is compared in Fig. 15. When the performance evaluation has a low belief degree and the expected utility fluctuates considerably, the perturbation coefficient is more likely to increase. This phenomenon indicates that when the indicator data fluctuate violently, it is more susceptible to the influence of perturbation conditions. Therefore, the rolling bearing should be adjusted or maintained to ensure its normal operation. Suppose that the maximum error of the perturbation coefficient is $\varepsilon = 0.4$. Obviously, when the perturbation intensity is 0.0015, 0.0030 and 0.0045, $|S_{i} | \le \varepsilon$ is constant, indicating that the rolling bearing has good adaptability to perturbation conditions. When the perturbation intensity is 0.0060, $|S_{4} | \le \varepsilon$ does not hold, indicating that the performance reliability of the rolling bearing is low and cannot work normally. At this time, the rolling bearing needs to be adjusted or maintained.

### Experiment 2

To further demonstrate the validity of the proposed method, we have added the frequency-domain indicators to the model.

#### Indicator data analysis

**Figure 16:**
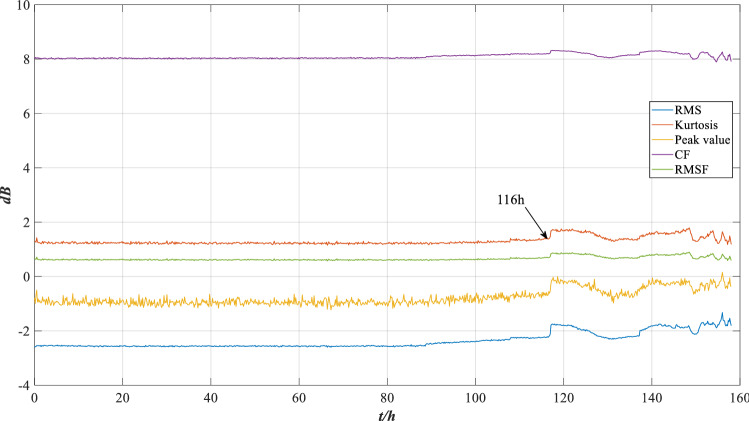
The trend of characteristic indicators.

The RMS, peak value, kurtosis, centroid frequency (CF) and root mean square frequency (RMSF) are extracted for analysis, as shown in Fig. 16.

As shown in Fig. 16, the RMS, peak value and kurtosis have obvious variation trends, the trend of centroid frequency and RMSF is not obvious. Therefore, the indicators of RMS, kurtosis and RMSF are selected for analysis in this section.

#### Performance evaluation of rolling bearing based on the ER rule

As in “Performance evaluation of rolling bearing based on the ER rule”, the identification frame is $\Theta = \{ (H_{1} ,high),(H_{2} ,medium),(H_{3} ,low)\}$. The reference values are shown in Table 4.

**Table 4:** Indicator evaluation level and reference value.

	$H_{1}$	$H_{2}$	$H_{3}$
RMS	− 2.604	− 2.234	− 1.325
Kurtosis	1.151	1.368	1.790
RMSF	0.576	0.612	0.895

**Figure 17:**
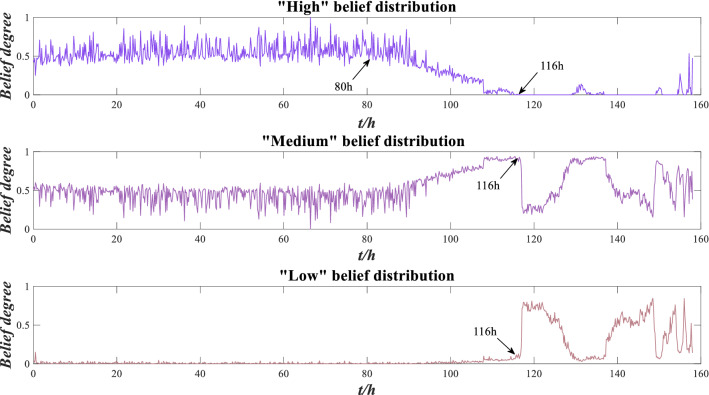
Performance evaluation results of rolling bearing.

Suppose the reliability of the RMS, kurtosis and RMSF are $r_{1}$, $r_{2}$ and $r_{3}$ respectively, and the weights are $\omega_{1}$, $\omega_{2}$ and $\omega_{3}$, respectively. According to Eqs. (14)–(16), the evidence reliabilities are $r_{1} = 0.2254$, $r_{2} = 0.2414$ and $r_{3} = 0.2414$, respectively. According to Eqs. (17)–(18), the evidence weights are $\omega_{1} = 0.3471$, $\omega_{2} = 0.3265$ and $\omega_{3} = 0.3265$, respectively. The ER rule is used to fuse the above indicator information according to Eqs. (19)–(30), and the evaluation results of rolling bearing performance are obtained as shown in Fig. 17.

As shown in Fig. 17, the evaluation results are mostly concentrated around the “high” belief degree, followed by the “medium” belief degree, and a few evaluation results are in the “low” belief degree, mainly distributed after 116 h, which is similar to Fig. 6. Before 80 h, the performance of the rolling bearing is good, and after 116 h, the bearing wear is serious.

**Figure 18:**
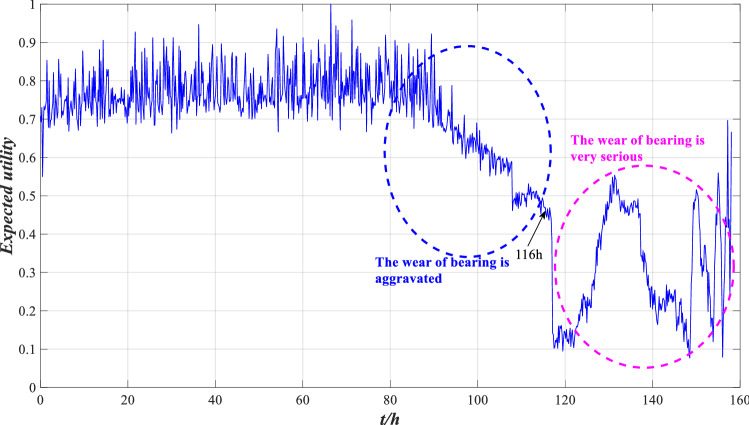
Expected utility of rolling bearing performance.

Suppose the utility of the reference grade is $u(H_{1} ) = 1$, $u(H_{2} ) = 0.5$ and $u(H_{3} ) = 0$, and the expected utility is calculated by Eq. (31), as shown in Fig. 18.

According to Fig. 18, the expected utility is distributed between 0.5 and 1 before 116 h, and the wear is aggravated between 80 and 116 h. After 116 h, the expected utility drops sharply and fluctuates sharply between 120 and 158 h, indicating that bearing wear is serious after 116 h. This is consistent with the results shown in Fig. 7, indicating that the model is still valid with different characteristic indicators.

**Figure 19:**
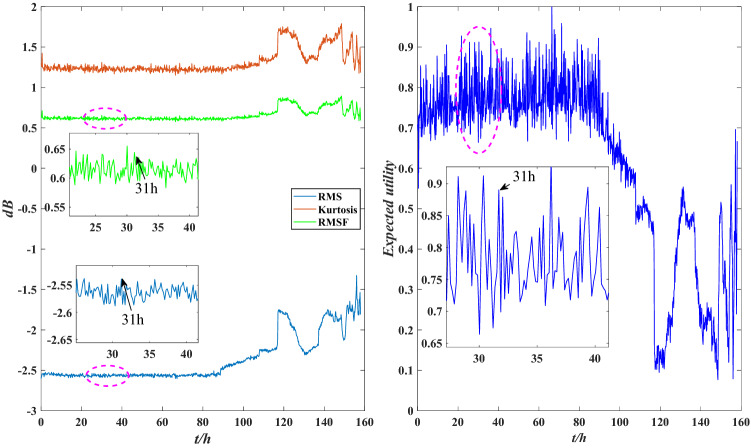
Local indicators and utility comparisons.

**Figure 20:**
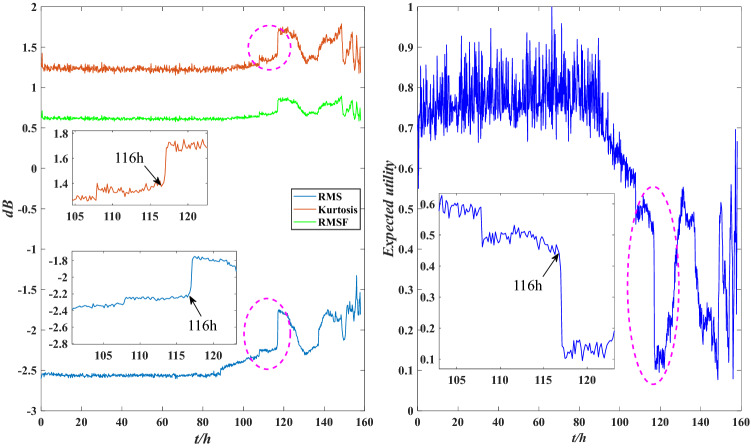
Local indicators and utility comparisons.

**Figure 21:**
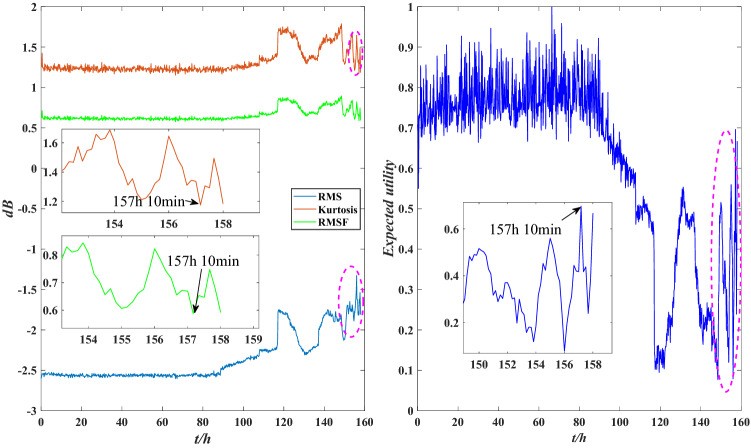
Local indicators and utility comparisons.

The comparison between the indicator data and the utility of the bearing under normal operation is shown in Fig. 19. The RMS and kurtosis values rise slightly at 31 h, and the utility shows a downtrend at this time, indicating that the stability of the performance is reduced. The comparisons between the indicator data and the utility of the abnormal operation are shown in Figs. 20 and 21. The RMS and kurtosis values increase sharply at 116 h, indicating that the stability of the rolling bearing performance decreases sharply. The RMSF and kurtosis value decrease slightly at 157 h 10 min, the bearing obtains some compensation which leads to an increase in its stability at this time, and the utility also shows an uptrend at 157 h 10 min. Therefore, the evaluation results of the model are valid.

#### Performance reliability analysis of rolling bearing considering perturbation

**Figure 22:**
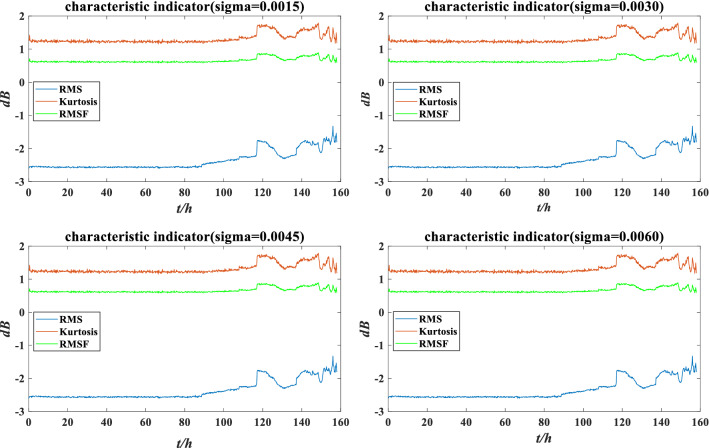
The characteristic indicators under different perturbation intensities.

Suppose the four perturbation environments are still low load and high sealing, low load and low sealing, high load and high sealing, high load and low sealing. The corresponding perturbation intensities are 0.0015, 0.0030, 0.0045 and 0.0060. The perturbation variable $\Delta x$ is shown in Fig. 11. After the addition of perturbation, each characteristic indicator data is changed. The distribution of indicator data under different perturbation intensities are listed in Fig. 22.

The reliability and weight of each indicator under different perturbation intensities are calculated according to Eqs. (14)–(18), as shown in Tables 5 and 6.

**Table 5:** Indicator reliability under different perturbation intensities.

$r$	$\sigma = 0.0015$	$\sigma = 0.0030$	$\sigma = 0.0045$	$\sigma = 0.0060$
RMS	0.2251	0.2247	0.2244	0.2241
Kurtosis	0.2419	0.2423	0.2428	0.2433
RMSF	0.2423	0.2433	0.2443	0.2454

**Table 6:** Indicator weight under different perturbation intensities.

$\omega$	$\sigma = 0.0015$	$\sigma = 0.0030$	$\sigma = 0.0045$	$\sigma = 0.0060$
RMS	0.3471	0.3471	0.3470	0.3469
Kurtosis	0.3265	0.3264	0.3264	0.3263
RMSF	0.3264	0.3265	0.3266	0.3268

**Figure 23:**
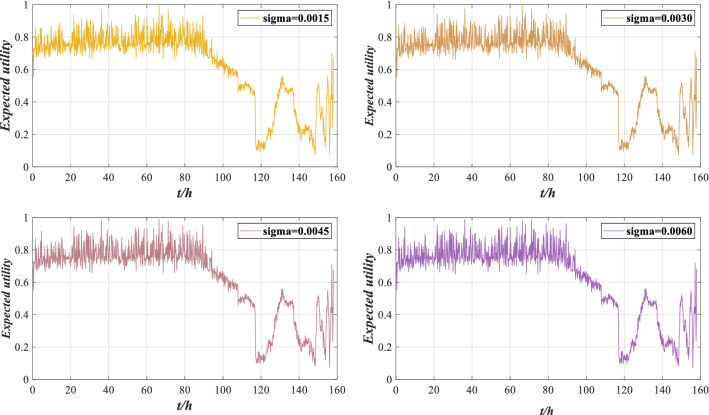
Expected utility of rolling bearing performance under different perturbation intensities.

Through utility calculation, the expected utility of rolling bearing performance reliability evaluation results under perturbation are as shown in Fig. 23.

According to Fig. 23, the evaluation results are similar to Fig. 13. The performance of the rolling bearing is good before 116 h, and the performance decreases after 116 h. This is consistent with the actual situation, indicating that the model can still accurately evaluate the performance of rolling bearing under perturbation.

### The difference comparison of characteristic indicators and utility

To demonstrate the advantages of using the ER rule to build the performance evaluation model, different characteristic indicators and expected utility are shown in this section.

**Figure 24:**
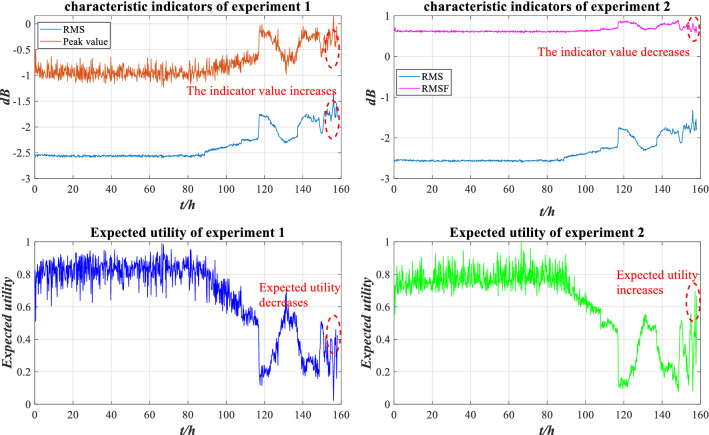
Comparison of characteristic indicators and expected utility.

According to Fig. 24, the peak value is used in Experiment 1, and its change trend is similar to RMS, with the indicator value increasing significantly between 150 and 158 h. The higher the indicator value is, the worse the bearing performance is, and the expected utility value is low. In experiment 2, the trend of RMSF is more gradual than that of RMS, and its indicator value is smaller between 150 and 158 h. When the bearing is worn to a certain extent, the abrasion is compensated in some ways to improve its performance. From 150 to 158 h, the expected utility is significantly larger than the expected utility in Experiment 1, which indicates that the evaluation result of the ER rule will be associated with the indicator trend. Therefore, the performance evaluation model of rolling bearing based on the ER rule is valid. In practical engineering, there are many monitoring indicators of complex systems with different characteristics. If only a single characteristic indicator is analysed, its health state cannot be comprehensively reflected. Therefore, selecting appropriate indicators is an important prerequisite for the evaluation of the ER rule.

### Comparative studies

The ER rule is essentially a kind of expert system. The expert experience and the objective data of actual engineering are effectively combined by the ER rule, and the evaluation results that consider both qualitative and quantitative aspects are obtained, which effectively reduces the subjectivity of expert experience and the objectivity of engineering data. To demonstrate the advantage of the ER rule, the analytic hierarchy process (AHP) and fuzzy expert system are used as comparative methods to compare with the ER model in this article.

#### AHP method

First, the discriminant matrix is established. The indicator system contains three indicators: effective value, peak value and kurtosis. The pairwise comparison method and $1\sim 9$ comparison method are adopted^35^, and the third-order discriminant matrix $A$ is given according to expert weighting:

35 $$ A{ = }\left[ {\begin{array}{*{20}c} 1 & { \, \frac{3}{2}} & { \, \frac{5}{4}} \\ \frac{2}{3} & { \, 1} & { \, \frac{3}{4}} \\ \frac{4}{5} & { \, \frac{4}{3}} & { \, 1} \\ \end{array} } \right] $$

To verify the rationality of the judgment matrix, it is necessary to conduct a consistency check on matrix $A$. Through eigenvalue calculation, it can be concluded that the maximum eigenvalue of $A$ is $\lambda_{\max } = 3.0012$, so the consistency test indicators are as follows:

36 $$ C_{I} = \frac{{\lambda_{\max } - n^{^{\prime}} }}{{n^{^{\prime}} - 1}} = \frac{3.0012 - 3}{{3 - 1}} \approx 0.000617 $$

where $n^{^{\prime}}$ is the order of the discriminant matrix $A$. As seen from the table, when the order is 3, the average random consistency index $R_{I} = 0.58$; then, the consistency ratio of matrix $A$ is as follows:

37 $$ C_{R} = \frac{{C_{I} }}{{R_{I} }} = \frac{0.000617}{{0.58}} \approx 0.0011 $$

Obviously, $C_{R} < 0.10$, so the discriminant matrix $A$ is reasonable. Then, the geometric average method is used to calculate the weight matrix of the index^35^, and the equation is as follows:

38 $$ W = \frac{w}{{\sum\nolimits_{i = 1}^{{n^{^{\prime}} }} {w_{i} } }},\;n^{^{\prime}} = 3 $$

39 $$ w_{i} = \sqrt[\begin{subarray}{l} n^{^{\prime}} \\ \\ \end{subarray} ]{{\prod\limits_{j = 1}^{{n^{^{\prime}} }} {w_{ij} } }} $$

$w$ is the row matrix of row 1 and $n^{^{\prime}}$ columns composed of $w_{i}$, $w_{ij}$ is the element in the discriminant matrix $A$, and $i,j = 1,2,3$. The weight matrix $W$ is obtained by Eqs. (38) and (39):

40 $$ W = [0.4045,0.2604,0.3352] $$

According to the indicator reference grade and reference value shown in Table 1, the membership degree of each indicator to the evaluation grade set is transformed into a fuzzy relation matrix^36^:

41 $$ M = \left[ {\begin{array}{*{20}c} {m_{11} } & {m_{12} } & {m_{13} } \\ {m_{21} } & {m_{22} } & {m_{23} } \\ {m_{31} } & {m_{32} } & {m_{33} } \\ \end{array} } \right] $$

where $m_{ij}$ is the membership degree of evaluation indicator $e_{i}$ relative to evaluation grade $H_{ij}$. By normalizing the reference values shown in Table 1, the fuzzy relation matrix can be obtained:

42 $$ M = \left[ {\begin{array}{*{20}c} {0.4225} & {0.3625} & {0.2150} \\ {0.7249} & {0.3672} & { - 0.0921} \\ {0.2672} & {0.3175} & {0.4153} \\ \end{array} } \right] $$

According to Eqs. (40) and (42), the evaluation results of rolling bearing performance can be obtained as follows:

43 $$ P = WM = [0.4492,0.3486,0.2022] $$

According to Eq. (43), the belief degree of the rolling bearing performance evaluated by AHP with respect to the “high”, “medium” and “low” grades are 0.4492, 0.3486 and 0.2022, respectively. The belief degree of the indicator data shown in Fig. 6 is mostly distributed in the “high” grade, and the result of the AHP method is also the largest value of the “high” grade, which indicates that AHP method is feasible in evaluating the performance of rolling bearing. However, the AHP method can only evaluate in general and cannot describe when the bearing’s performance deteriorates and changes, so the evaluation results cannot effectively describe the actual situation. In addition, when the AHP method is used to perform performance, the parameter matrix set will be affected by the subjective uncertainty of experts, and the evaluation results cannot objectively describe the data.

#### Fuzzy expert system

**Figure 25:**
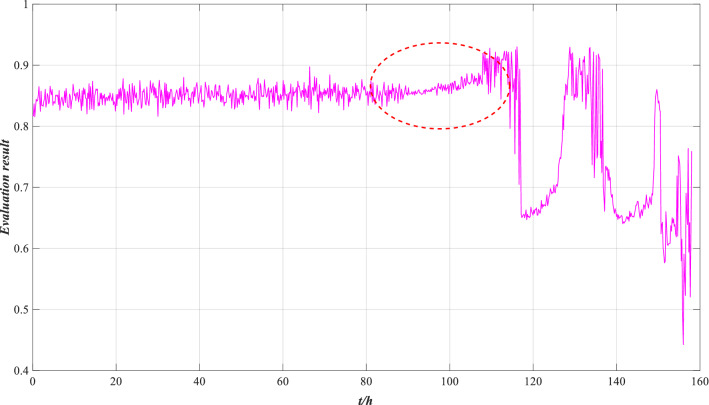
Evaluation result of the fuzzy expert system.

A fuzzy expert system is a knowledge-driven method that expresses knowledge by rules. It can effectively deal with uncertain data^37^. According to expert knowledge, nine kinds of performance state grades of the rolling bearing are set, and the performance state of the rolling bearing is evaluated by calculating the membership degree of each grade. The evaluation results are shown in Fig. 25.

As shown in Fig. 25, compared with the AHP method, the fuzzy expert system can evaluate the performance state of rolling bearing in real time. However, the wear of rolling bearing increased between 80 and 116 h, and the evaluation result showed an uptrend, which is inconsistent with the actual situation. In essence, the fuzzy expert system relies on expert knowledge to set evaluation rules, which is too subjective. If the rules are not set reasonably, the evaluation result will be biased, so the method cannot effectively evaluate the performance of the rolling bearing.

In conclusion, the AHP method cannot evaluate performance in real time. A fuzzy expert system relies on expert knowledge, if the expert knowledge is unreasonable, the evaluation result will be unreasonable. Therefore, the ER evaluation model proposed in this article fully considers expert knowledge and engineering data, reflecting the advantages of the ER rule in dealing with uncertainty, so the performance evaluation model constructed in this article is reasonable.

### Application of the evaluation model to XJTU-SY bearing data set

To demonstrate the universality of the proposed evaluation models, we applied the models to the rolling bearing data set of Xi’an Jiaotong University. This data set is provided by the Institute of Design Science and Basic Component at Xi’an Jiaotong University (XJTU), and the Changxing Sumyoung (SY) Technology^38^.

#### Data analysis

In the test platform, the AC motor rotates at a constant speed of 2250 RPM and is connected to the shaft through a friction belt. Five rolling bearings are installed on the shaft. The vibration signal data were collected sampling frequency is 25.6 kHz and are collected at an interval of 1 min for one second, and a total of 32,768 points are collected. The data collected in bearing 5 are used as experimental data, and test time is 339 min.

**Figure 26:**
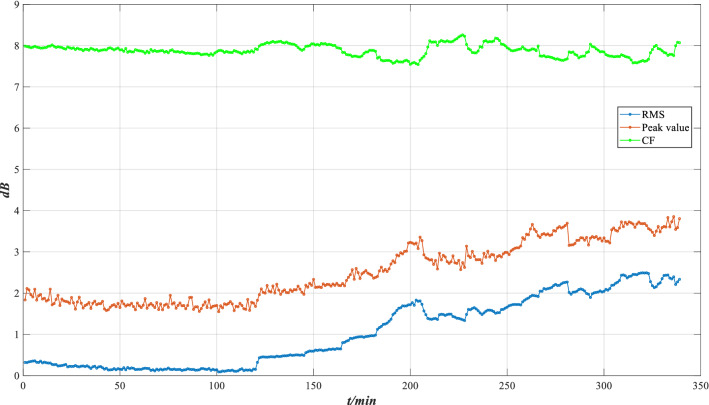
Characteristic indicators of XJTU-SY bearing.

Based on Eqs. (5), (6) and (11), the characteristic indicators of RMS, peak value and CF are shown in Fig. 26.

As shown in Fig. 26, as the operation time increases, the RMS and the peak value indicator gradually increase, and the CF indicator fluctuates irregularly. At 120 min, the RMS and the Peak value gradually increase and reach the peak at 205 min. After 205 min, the RMS and the Peak value generally show an uptrend with fluctuations, and the CF shows an irregular fluctuation trend. This indicates that the bearing’s performance decreases as time goes. Moreover, to ensure that the indicator value is greater than 0, all the indicator values are increased by 0.2.

#### Performance evaluation of the XJTU-SY bearing based on the ER rule

Suppose the identification frame is $\Theta = \{ (H_{1} ,high),(H_{2} ,medium),(H_{3} ,low)\}$. The reference values are shown in Table 7. The “high” and “low” reference values of RMS, peak value and CF are the minimum value and maximum value of indicator data, respectively, and since the bearing wear is aggravated at 200–250 min, the “medium” reference value is the denser point of data distribution between 200 and 250 min.

**Table 7:** Indicator evaluation level and reference value.

	$H_{1}$	$H_{2}$	$H_{3}$
RMS	0.090	1.598	2.488
Peak value	1.551	2.837	3.850
CF	7.544	8.093	8.257

**Figure 27:**
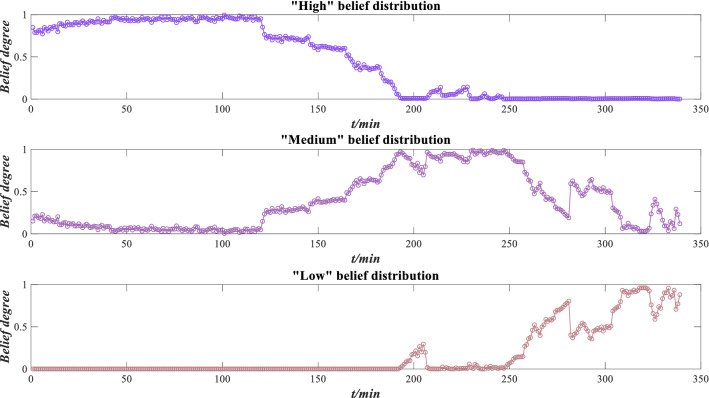
Performance evaluation results of XJTU-SY bearing.

Based on Eqs. (14)–(16), the evidence reliabilities are $r_{1} = 0.5275$, $r_{2} = 0.4804$ and $r_{3} = 0.2949$, respectively. Based on Eqs. (17)–(18), the evidence weights are $\omega_{1} = 0.7200$, $\omega_{2} = 0.2633$ and $\omega_{3} = 0.0167$, respectively. The ER rule is used to fuse this information by Eqs. (19)–(30), and the performance evaluation results are obtained in Fig. 27.

According to Fig. 27, the “high” belief degree plays a more significant role before 120 min, the “medium” belief degree reaches its peak around 190 min, and the “low” belief degree shows an uptrend after 250 min. This indicates that with the increase of operation time, the bearing wear is aggravated, which is consistent with the variation trend of bearing capacity in Fig. 26.

**Figure 28:**
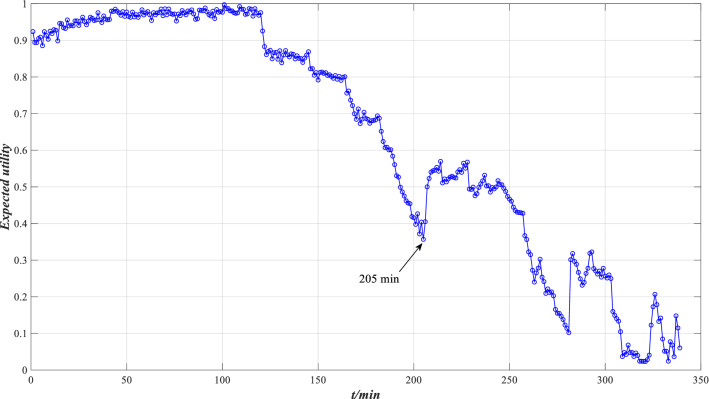
Expected utility of XJTU-SY bearing performance.

Suppose the utility of the reference grade is $u(H_{1} ) = 1$, $u(H_{2} ) = 0.5$ and $u(H_{3} ) = 0$, and the expected utility of performance is calculated by Eq. (31), as shown in Fig. 28.

According to Fig. 28, the change in bearing’s performance shows a downtrend after 120 min, but there are some brief uptrends in the middle. After 205 min, the expected utility begins to increase greatly, indicating that the bearing’s performance begins to rise due to some compensation. This is consistent with the indicator trend in Fig. 26.

#### Performance reliability analysis of XJTU-SY bearing considering perturbation

**Figure 29:**
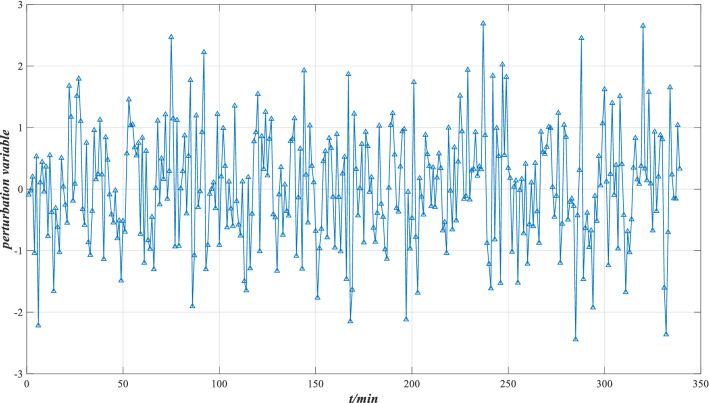
Perturbation variable distribution.

**Figure 30:**
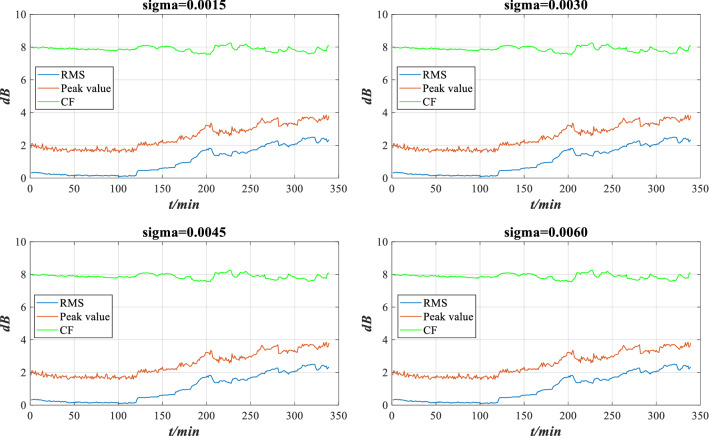
The characteristic indicators of XJTU-SY bearing under different perturbation intensities.

Suppose perturbation intensities are 0.0015, 0.0030, 0.0045 and 0.0060. The distribution of perturbation variable $\Delta x$ is shown in Fig. 29. The indicator data under different perturbation intensities are listed in Fig. 30.

Due to the perturbation has little influence on the indicator data, and the weight change of the indicator can be ignored. Therefore, based on the Eqs. (17) and (18), the bearing weights are $\omega_{1} = 0.7200$, $\omega_{2} = 0.2633$ and $\omega_{3} = 0.0167$, respectively. The bearing reliability under different perturbation intensities are calculated by Eqs. (14)–(16), as shown in Tables 8.

**Table 8:** Reliability under different perturbation intensities.

$r$	$\sigma = 0.0015$	$\sigma = 0.0030$	$\sigma = 0.0045$	$\sigma = 0.0060$
RMS	0.5262	0.5248	0.5234	0.5220
Peak value	0.4805	0.4807	0.4808	0.4809
CF	0.2951	0.2953	0.2955	0.2957

**Figure 31:**
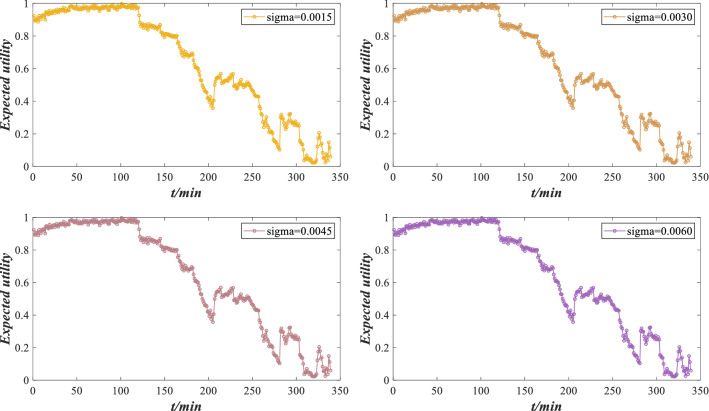
Expected utility of XJTU-SY bearing performance under different perturbation intensities.

The expected utility of XJTU-SY bearing performance reliability evaluation results under perturbation are shown in Fig. 31.

**Figure 32:**
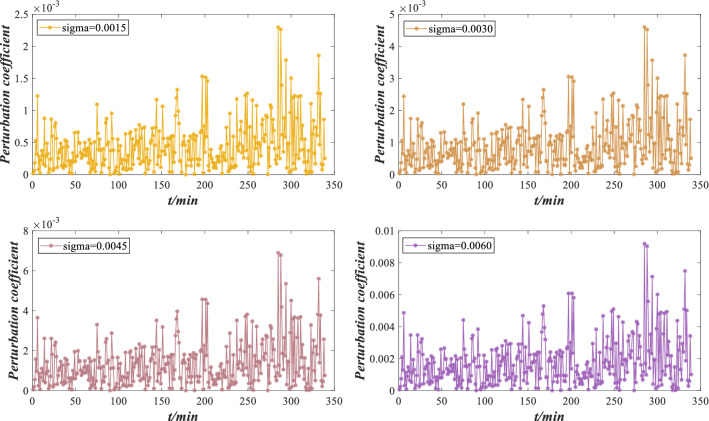
Perturbation coefficients under different perturbation intensities.

According to Fig. 31, there are slight deviations in the performance under different perturbations, but the overall evaluation result is similar to the utility without perturbation. The model can effectively evaluate bearing performance under perturbation. The perturbation coefficients under different perturbation intensities are shown in Fig. 32.

According to Fig. 32, with the increase in perturbation intensity, the perturbation coefficient also increases, which is consistent with the negative influence of perturbation on the bearing. When the utility has a large fluctuation, the perturbation coefficient also increases. After 205 min and 281 min, the bearing performance fluctuates seriously, and the perturbation coefficient increases sharply. This indicates that when bearing performance is unstable, it is more susceptible to perturbation. Therefore, it is essential to adjust or maintain the bearing to ensure its operation. Suppose that the maximum error of the perturbation coefficient is $\varepsilon = 0.008$. When the perturbation intensity is 0.0015, 0.0030 and 0.0045, $|S_{i} | \le \varepsilon$ is constant, indicating that the bearing has good adaptability to perturbation. When the perturbation intensity is 0.0060, $|S_{4} | \le \varepsilon$ does not hold, indicating that the performance reliability of the bearing is low and cannot work normally. At this time, the bearing needs to be adjusted or maintained.

## Conclusion

Based on analysing the characteristics of rolling bearing, the indicator evaluation system based on vibration signal is innovatively used, and a performance evaluation model of rolling bearing based on the ER rule is proposed. This is used to evaluate the bearing performance under ideal working conditions. Then, a performance reliability evaluation model considering perturbation is established, by adding a perturbation analysis to simulate the influence of different perturbations on rolling bearing. The time domain indicators and frequency domain indicators are used to construct the evaluation system. The utility-based method is used to unify the indicator information into the form of a belief distribution, which improves the expression ability. The distance-based method and COV method are used to determine the reliability and weight of indicators, which overcome the subjectivity of expert knowledge weighting and improve the reliability of performance evaluation results. Finally, the rolling bearing adaptability to the perturbations is quantified by using the perturbation coefficient and the maximum error. The experimental results show that the model has a good effect on the performance and reliability analysis of rolling bearing. Moreover, the models are applied to the performance evaluation of XJTU-SY bearing, and obtain effective results.

The main work in the future will include the following aspects: (1) When setting the reference values, if the input data exceed the evidence range, then the adaptive method is supposed to be used to determine the reference values. Therefore, building an adaptive method will be the focus of future work. (2) The evaluation model based on the ER rule can be used to discuss the performance of rocket structures, high bridges, and other complex systems. Because this system may often face perturbations, the ER rule can also be extended to other fields for evaluation and decision-making.

## Data Availability

The experimental data in this article come from the bearing datasets of the Kaggle platform and Google drive. For the source URL of the bearing data set of the University of Cincinnati, please visit: https://www.kaggle.com/datasets/vinayak123tyagi/bearing-dataset. For the source URL of the XJTU-SY bearing data set, please visit: https://drive.google.com/drive/folders/1ueg67JZcIoAM6KiOz1a4XDkh6lh2Y8Ii.
